# Assessment of Biological Activities of Fungal Endophytes Derived Bioactive Compounds Isolated from *Amoora rohituka*

**DOI:** 10.3390/jof8030285

**Published:** 2022-03-10

**Authors:** Ashish Verma, Priyamvada Gupta, Nilesh Rai, Rajan Kumar Tiwari, Ajay Kumar, Prafull Salvi, Swapnil C. Kamble, Santosh Kumar Singh, Vibhav Gautam

**Affiliations:** 1 Centre of Experimental Medicine and Surgery, Institute of Medical Sciences, Banaras Hindu University, Varanasi 221005, India; ashishambbhu@gmail.com (A.V.); priyamvada17gupta@gmail.com (P.G.); nilesh.rai17@bhu.ac.in (N.R.); singhsk71@yahoo.com (S.K.S.); 2 Department of Zoology, Institute of Science, Banaras Hindu University, Varanasi 221005, India; shandilya.raj31@gmail.com (R.K.T.); ajayzoo@bhu.ac.in (A.K.); 3 Department of Agriculture Biotechnology, National Agri-Food Biotechnology Institute, Sahibzada Ajit Singh Nagar 140306, India; prafull.salvi@nabi.res.in; 4 Department of Technology, Savitribai Phule Pune University, Pune 411007, India; sckamble@unipune.ac.in

**Keywords:** *Amoora rohituka*, fungal endophyte, *Penicillium oxalicum*, antioxidant activity, cytotoxic activity

## Abstract

Fungal endophytes have remarkable potential to produce bioactive compounds with numerous pharmacological significance that are used in various disease management and human welfare. In the current study, a total of eight fungal endophytes were isolated from the leaf tissue of *Amoora rohituka*, and out of which ethyl acetate (EA) extract of *Penicillium oxalicum* was found to exhibit potential antioxidant activity against DPPH, nitric oxide, superoxide anion and hydroxyl free radicals with EC_50_ values of 178.30 ± 1.446, 75.79 ± 0.692, 169.28 ± 0.402 and 126.12 ± 0.636 µg/mL, respectively. The significant antioxidant activity of EA extract of *P. oxalicum* is validated through highest phenolic and flavonoid content, and the presence of unique bioactive components observed through high-performance thin layer chromatography (HPTLC) fingerprinting. Moreover, EA extract of *P. oxalicum* also displayed substantial anti-proliferative activity with IC_50_ values of 56.81 ± 0.617, 37.24 ± 1.26 and 260.627 ± 5.415 µg/mL against three cancer cells HuT-78, MDA-MB-231 and MCF-7, respectively. Furthermore, comparative HPTLC fingerprint analysis and antioxidant activity of *P. oxalicum* revealed that fungal endophyte *P. oxalicum* produces bioactive compounds in a host-dependent manner. Therefore, the present study signifies that fungal endophyte *P. oxalicum* associated with the leaf of *A. rohituka* could be a potential source of bioactive compounds with antioxidant and anticancer activity.

## 1. Introduction

Medicinal plants have been a potential therapeutic and curative agent for a wealth of diseases and healthcare attributed to the phytochemicals constituted in them. The command of plants in the pharmacological evaluation is ascribed to the secondary metabolites, flavonoids, terpenoids, tannins and alkaloids [1]. It is estimated by WHO (World Health Organization) that about 80 percent of the world’s population depend upon herbal plants for their natural products by virtue of their flexibility, low cost, easy availability, better compatibility with the human body, and ethical acceptability with minimal negative effect [1,2]. In contemplation of conservation, sustainable use and protection of ethnobotanical aspects of plants, researchers have been heading towards the isolation of plant associated microbial community for the extraction of bioactive compounds similar to plants. The fungal endophytes meet this demand and serve as a true alternative of plant-derived bioactive compounds. The establishment of fungal relationship with the host plant can vary from symbiotic to commensalism, and can also be parasitic [3]. The symbiotic ones during the process of co-evolution have started synthesizing the compounds similar to their host and showed therapeutic potential similar to the host’s compound [4]. Endophytes have a great prospect for the production of a broad spectrum of secondary metabolites and these natural products have been reported to exhibit various biological activities [5]. The versatile synthetic tendency of fungi confers extrapolation of an extensive range of metabolites. The fungal endophyte derived metabolites are natural, affordable and less-toxic, showing a multifunction mechanism that labels them as distinctive entities. The fungal endophytes serve as a reservoir of terpenoids, phenolic acids, quinones, tannins, alkaloids, saponins, and steroids, and therefore, fungal endophyte-derived bioactive compounds display an extensive range of activities in human health and diseases, including antibiotic, antifungal, immunomodulatory, antioxidant, antidiabetic and anticancer [6,7]. A wide range of bioactive compounds have been isolated from fungal endophytes and have been studied for their chemistry using omics and mass spectrometry-based tools [8]. On account of these properties, fungal endophytes appear as an epitome of natural products with their endowment in the field of agriculture, industry and pharmaceuticals.

*Amoora rohituka* (*Aphanamixis polystachya*), also known as Pithraj tree, is a 20–30 m tall deciduous tree belonging to the Meliaceae family and is native to India. It is one of the eminent plants with pronounced medicinal properties. The seeds and stem bark have been shown to have a significant role in the treatment of tumor, splenomegaly and liver disorders [9]. The alcoholic extract of stem bark has shown significant anticancer activity against Ehrlich ascites carcinoma and Friend’s leukemia in mice [10,11,12]. Another report has been obtained for radiation-induced chromosome damage by ethyl acetate fraction of *A. rohituka* [13]. A bioactive compound rohitukine extracted from *A. rohituka* possesses immune-modulatory, anti-inflammatory and anti-cancer properties, and also serves as a precursor for the synthesis of P-276-00 (Piramal Healthcare Limited, Mumbai, India) and flavopiridol, an anticancer drug [14,15]. The first study for isolation of rohitukine and its analog, rohitukine N-oxide has been demonstrated in endophytic fungi *Gibberella fujikuroi* MTCC 11382 obtained from *A. rohituka* [16]. In other reports, the compound rohitukine extracted from a tree *Dysoxylum binectariferum* have been shown to possess immunomodulatory, anti-inflammatory activities [17] and also reported for attenuating peptic ulcers in rat [18]. Moreover, cytotoxic activity of rohitukine has been reported in colorectal cancer and promyelocytic leukemia [19]. Previous studies have shown leaf extract of *A. rohituka* exhibit potential anticancer activity against human breast cancer cell (MCF-7) [20]. In another study, it has been reported that leaves extract of *A. rohituka* possess significant antioxidant, cytotoxic and thrombolytic activities [21]. The aforementioned studies have been performed for the isolation and characterization of natural compounds either from plant *A. rohituka* or its part including root, stem bark, leaf and seeds against various diseases including cancer. However, this approach leads to the overexploitation of medicinal plants, and therefore a better alternative is needed. The properties of fungal endophytes such as easy cultivation, scale up flexibility, high yield and unique cellular organization offer them as an excellent alternative to isolate bioactive compounds similar to host plant metabolites with numerous pharmacological properties [4]. Considering such advantages of fungal endophytes over plant tissue, the leaf of *A. rohituka-*associated fungal endophytes was assessed for the production of bioactive compounds with antioxidant and cytotoxic activity. An increasing body of evidence suggests that leaf tissue is the most prevalent part to be inhabited by fungal endophytes due to its large surface area and being more exposed to the environmental factors [5,6,7]. The selection of leaf tissue provides leaves of diverse stages that could be young leaf, premature leaf and mature leaf and that show an association of a wide range of fungal endophytes.

The aforementioned fact compelled us towards the isolation of fungal endophytes associated with the leaf tissue of *A. rohituka*, and further to evaluate the antioxidant and cytotoxic activity of bioactive compounds present in the EA extract of isolated fungal strains. Total phenolic and flavonoid content and HPTLC fingerprinting were performed for the qualitative screening of bioactive constituents produced by the identified fungal endophytes. The antioxidant activity of fungal endophyte derived bioactive compounds was evaluated against free radicals through DPPH free radical scavenging assay, superoxide anion scavenging assay, hydroxyl radical scavenging assay and nitric oxide scavenging assay. Moreover, based on the results of biochemical profiling and antioxidant activity of isolated fungal strains, EA extract of *P. oxalicum* was further selected to investigate the cytotoxic activity against human-derived cancer cells, T-cell lymphoma cell (HuT-78) and breast cancer cells (MDA-MB-231 and MCF-7). Host-dependent activity and production of bioactive compounds derived from fungal endophyte was also evaluated through performing the antioxidant activity and HPTLC fingerprinting profile of EA extract of fungal endophyte *P. oxalicum* isolated from the leaf of *A. rohituka* and rhizospheric soil of maize. Conclusively, our findings suggest the presence of potential antioxidant and cytotoxic compounds in EA extract *P. oxalicum* isolated from the leaf of *A. rohituka.*

## 2. Materials and Method

### 2.1. Collection of Plant Sample and Isolation of Endophytic Fungi

Fungal endophytes were isolated from the healthy leaves of plant *A*. *rohituka*, collected from the Department of Dravyaguna, Institute of Medical Sciences, Banaras Hindu University, Varanasi, Uttar Pradesh (India). The collected leaf samples were washed and surface disinfected according to the method discussed earlier [22]. Briefly, the leaf samples were rinsed with running tap water followed by deionized water and subsequently dipped in 70% ethanol (1–2 min) followed by sterilization in 0.1% sodium hypochlorite (2–3 min). Then, they were further dipped in 70% ethanol and finally rinsed with distilled water. Dried leaves were allowed to dry, cut aseptically into small pieces (1 cm^2^) and patched onto potato dextrose agar (PDA) (Himedia, Mumbai, India) plates containing streptomycin (SRL, Mumbai, India) at a concentration of 250 μg/mL to prevent bacterial contamination. The effectiveness of surface sterilization was tested by examining the growth of epiphytes by spreading the water onto PDA plates obtained after last wash. PDA plates were placed in BOD incubator (Narang Scientific Works Pvt. Ltd., New Delhi, India) at 27 ± 2 °C for 7–10 days. The fungal mycelia emerging from plant parts were isolated following subculturing of the mycelia from different fungi onto fresh PDA plates until appearance of a pure individual colony.

### 2.2. Morphological and Molecular Identification of Isolated Fungal Strains

The morphological characterization of isolated fungal isolates was done through the preparation of semi-permanent slides of fungal mycelia. For the semi-permanent slide preparation, a fungal colony was picked from PDA plate by sterile needle and placed on a glass slide, and then Lacto-phenol cotton blue was added for staining, and observed under bright field microscope (Olympus, CX43, Tokyo, Japan) [23]. For molecular identification, the genomic DNA (gDNA) was isolated from fungal mycelia using Nucleo-pore gDNA Fungal/Bacterial mini kit followed according to the manufacturer’s protocol. After extraction, conserved ITS region of fungal gDNA was amplified by general primers ITS4 (5′-TCCTCCGCTTATTGATATGC-3′) and ITS5 (5′-GGAAGTAAAAGTCGTAACAAGG-3′). PCR reaction mixture contained 2 µL of extracted gDNA, 1.5 µL of each forward primer and reverse primer (10 µM concentration), 2 µL of 10× buffer, 0.5 μL of Taq DNA polymerase enzyme (BR Biochem, New Delhi, India), 0.75 μL of 10 mM of deoxynucleotide triphosphates (BR Biochem) and volume was maintained upto 20 μL with milli-Q water for single reaction of PCR. The PCR reaction was performed under the following condition: 5 min for initial denaturation step at 94 °C followed by 35 cycles at 94 °C for 30 s, annealing at 60 °C for 40 s and extension at 74 °C for 1 min and final extension step was for 10 min at 74 °C. After PCR cycle completion, gel electrophoresis was performed to examine the PCR products (Figure S1) by using 1% agarose gel in 1× TAE buffer. The PCR products so obtained were purified using Nucleopore Quick PCR purification kit as per manufacturer’s protocol, and sequencing of purified PCR product was performed using ITS4 and ITS5 primers.

### 2.3. Phylogenetic Tree Construction

The identification of fungal endophytes was done based on consensus DNA sequences. Homology searches of common sequences were performed through Basic Local Alignment Search Tool (BLAST) on http://www.ncbi.nlm.gov/BLAST (accessed on 21 November 2021) in NCBI database [24]. The identification of fungal endophytes and their consensus common DNA sequences was done by comparing query sequences with the previously submitted sequences in GenBank to obtained the accession number. Both FP-ITS and RP-ITS sequences were used separately where common overlapping sequence were not available. For the comparison and alignment of query sequences and reference sequences, MUSCLE (Multiple Sequence Comparison by Log-Expectation) program was used. The study of percentage identity of aligned sequences was done using Kolmogorov–Smirnov statistical test in GeneDoc (version 2.7). Using the obtained sequences, phylogenetic analysis was performed and a phylogenetic tree was constructed through MEGA (v10.1.8) by maximum likelihood Bootstrap (MLBS) method.

### 2.4. Fermentation Procedure and Extraction of Crude Fungal Extract

After morphological and molecular identification of fungal isolates, the grown PDA culture of individual fungal strain was inoculated into 300 mL potato-dextrose broth in a separate Erlenmeyer flask supplemented with streptomycin (250 μg/mL) and incubated at 27 ± 2 °C for 21 days at 140 rpm. After fermentation, the mycelium was harvested through filtration using the two-layered cheese cloth and was subjected to drying overnight at 50 °C. The dried mycelium so obtained was macerated individually with liquid nitrogen and fungal metabolites were extracted using ethyl acetate (5× volume of dry weight of mycelia) as a solvent. The crude fungal extract containing the bioactive compounds was stored at 4 °C for further experimental process.

### 2.5. Estimation of Total Flavonoid Content and Total Phenolic Content of EA Extract of Fungal Mycelia

The colorimetric determination of total flavonoid content was done by the AlCl_3_ method as described earlier [25]. The method involves mixing of 1 mL of EA extract isolated fungal endophytes with 3 mL methanol followed by 200 µL of AlCl_3_ (10%) and 200 µL of potassium acetate (9.8%). Further, the reaction mixture was diluted using 5.6 mL of distilled water and incubated for 30 min. Then, the absorption was taken at 420 nm against methanol, taken as a blank. Quercetin (5–200 μg/mL) was used to plot the calibration curve. The results obtained for total flavonoid content were expressed as µg of quercetin equivalents (QE) per mg of EA extract.

The Folin–Ciocalteu method was used for the determination of total phenolic content with some modifications [26]. Briefly, the sample (1 mL) was oxidized with 100 µL of the 0.5 N FC reagent and incubated for 15 min. Further, 2.5 mL of sodium carbonate (7.5%, *w*/*v*) was added to neutralize the reaction mixture and was mixed thoroughly. Following incubation for 30 min, absorbance was recorded at 727 nm against methanol, taken as a blank. The calibration curve was constructed using Gallic acid (5–200 μg/mL), and the results for total phenolic content was shown as µg of gallic acid equivalents (GAE) per mg of EA extract.

### 2.6. HPTLC Fingerprinting Analysis-Based Metabolites Profiling of EA Extract of Fungal Mycelia

The EA extract of fungal endophytes was screened for their bioactive compounds through CAMAG HPTLC equipment (ANCHROM, Muttenz, Switzerland) composed of a Linomat-4 autosampler, CAMAG TLC scanner-4 and visualizer. The EA extract of fungal endophyte was applied as bands on the plate through the software-based applicator. The mobile phase used for the development of the silica gel 60 F_254_ TLC plate (Merck, Darmstadt, Germany) was toluene: chloroform: ethyl alcohol (4:4:1, *v*/*v*/*v*). After application, the TLC plate was kept in Twin trough chamber CAMAG (pre-saturated with mobile phase for 30 min) for the chromatogram development up to 70 mm from lower edge of the plate. The plate was dried and images were captured using visualizer at short UV range 254 nm and long UV range 366 nm. Scanning of the TLC plate was done by TLC scanner-4 at 254 nm (D2 light source) and 366 nm (Hg light source), and analysis of area percentage and retention factor for the separated components was analyzed by winCATS Planar Chromatography Manager (version 1.4.10.0001). The separated components on the TLC plate after development were subjected to post-chromatographic derivatization using Anisaldehyde Sulphuric Acid reagent (ASR).

### 2.7. Antioxidant Assays

Antioxidant activity of isolated fungal extract was assessed using DPPH free radical scavenging assay, superoxide anion scavenging assay, hydroxyl radical scavenging assay and nitric oxide scavenging assay. For each extract EC_50_ value was calculated using Graph Pad Prism 8.0.2 software and data for antioxidant assays was analyzed for statistical significance using One-Way ANOVA followed by Tukey to determine statistical significance.

#### 2.7.1. Free Radical Scavenging Assay

The free radical scavenging activity of EA extract of fungal isolates was evaluated by colorimetric assay using DPPH as the source of free radical. Briefly, a fresh 50 μg/mL of DPPH solution (prepared in methanol, SRL, India) was added to different concentrations of EA extracts (1, 5, 10, 25, 50, 100 and 200 μg/mL) of fungal endophyte, vortexed vigorously and incubated for 30 min in dark at room temperature as described earlier [27]. After incubation, the absorbance was measured at 517 nm using UV-Vis spectrophotometer. The percentage of radical scavenging potential was calculated using the formula = [1 − (Abs₍_517nm_₎ of the sample/Abs₍_517nm_₎ of the control)] × 100. Ascorbic acid was used as a positive control and methanol was used as a blank.

#### 2.7.2. Superoxide Anion Scavenging Activity

To a mixture of 1 mL of 150 μM Nitroblue Tetrazolium solution (SRL) and 1 mL NADH (468 μM in 100 mM PBS, SRL, India) 200 μL of different concentrations (1, 5, 10, 25, 50, 100 and 200 μg/mL) of EA extract of each fungal endophyte were added. Reaction was started by adding 200 μL of phenazine methosulphate (60 mM PMS in 100 mM phosphate buffer, pH 7.4) solution and incubated at 25 °C for 5 min [28]. The absorbance was taken at 560 nm was measured against methanol as a blank. The percentage of superoxide anion radical scavenging potential was calculated using the formula = [1 − (Abs₍_560nm_₎ of the sample/Abs₍_560nm_₎ of the control)] × 100. Ascorbic acid was used as a positive control and methanol as a blank.

#### 2.7.3. Hydroxyl Radical Scavenging Assay

The different concentrations of EA extract of fungal isolate were added to a mixture of 100 μM FeCl_3_ (Merck), 100 mM EDTA, 3.75 mM 2-deoxyribose (SRL, India), and 1 mM H_2_O_2_ (Qualigens Fine Chemicals, Maharashtra, India). After incubation for 1 h at 37 °C, 1 mL of 2% Trichloroacetic acid (TCA) and 1% Thiobarbituric acid (TBA) were added to the reaction mixture and again incubation was done for 15 min at 90 °C boiling water-bath, and then absorbance was taken at 535 nm. The calculation for the percentage hydroxyl radical scavenging potential was done using the formula = [1 − (Abs₍_535nm_₎ of the sample/Abs₍_535nm_₎ of the control)] × 100 [27]. The positive control used was ascorbic acid and methanol was used as a blank.

#### 2.7.4. Nitric Oxide Scavenging Assay

Different concentrations (1, 5, 10, 25, 50, 100 and 200 μg/mL) of EA extract of fungal endophyte is mixed with 150 μL of (10 mM) sodium nitroprusside and following incubation of 150 min, the reaction mixture was added with 200 μL of Greiss reagent (SRL, India) and left for 30 min. The absorbance was then recorded at 546 nm. Ascorbic acid was used as a positive control and methanol was used as a blank. The percentage of nitric oxide scavenging potential was calculated using the formula = [1 − (Abs₍_546nm_₎ of the sample/Abs₍_546nm_₎ of the control)] × 100 [29].

### 2.8. Culture Media Preparation and Cell Line Maintenance

Two adherent human breast cancer cell lines MDA-MB-231 and MCF-7 and one suspension cell line of human malignant T-cells (HuT-78) were used to evaluate the cytotoxic effect of EA extract of *P. oxalicum*. HuT-78 cells were grown in 10% FBS containing Roswell Park Memorial Institute (RPMI) medium supplemented with 1% penicillin-streptomycin-amphotericin B solution (CELL clone) at 37 °C in a 5% CO_2_ incubator (Eppendorf, Darmstadt, Germany). MDA-MB-231 and MCF-7 cells were grown in Dulbecco’s modified Eagle’s medium (DMEM) supplemented with 10% fetal bovine serum (HI-FBS) (US grade, Thermo), and 1% penicillin-streptomycin-amphotericin B solution (CELL clone) and maintained at 37 °C in a 5% CO_2_ incubator. The cells were observed at every 24 h for cell growth and the presence of any contaminants.

#### Cytotoxicity Assay

The in vitro cytotoxic activity of EA extract of *P. oxalicum* was evaluated against HuT-78, MDA-MB-231 and MCF-7 cells based on the formation of insoluble formazan salt through reduction of 3-(4,5-dimethylthiazol-2-yl)-2,5-diphenyl tetrazolium bromide (MTT) by NAD(P)H-dependent cellular oxidoreductase enzymes that corresponds to viable cells remained after treatment with EA extract. The tumor cells (5 × 10^4^ cells/ well) were seeded in 96-well culture plates for 24 h at 37 °C in a humidified CO_2_ incubator. EA extract of *P. oxalicum* was dissolved in DMSO, and further stock was prepared through serial dilution with DMEM media. Tumor cells were treated with different concentrations of EA extract of *P. oxalicum* (0–200 µg/mL) for 24 h. Cells treated with 0.1% DMSO was considered as control. Thereafter, 20 μL of MTT (5 mg/mL, SRL, India) was added to each well and incubated for 4 h at 37 °C in a 5% CO_2_ incubator (Panasonic, Sakata, Japan). After 4 h, the plates were centrifuged for 20 min at 3000 rpm followed by solubilization of formazan crystals using 100 μL of DMSO. Absorbance was recorded at 570 nm using Multiskan™ FC microplate photometer (ThermoFisher Scientific, Waltham, MA, USA).

### 2.9. Statistical Analysis

The experiments of antioxidant assays and cytotoxic assay were performed in triplicate (*n* = 3). Data are presented as mean ± S.D. in histogram. For the statistical significance of antioxidant and cytotoxic data, One-Way ANOVA (analysis of variance) followed by Tukey to determine was performed using Graph Pad Prism 8.0.2 software and mean ± S.D. of all groups were compared.

## 3. Results and Discussion

### 3.1. A Total of 8 Fungal Endophytes Were Isolated Using Culture-Dependent Approach

**Figure 1:**
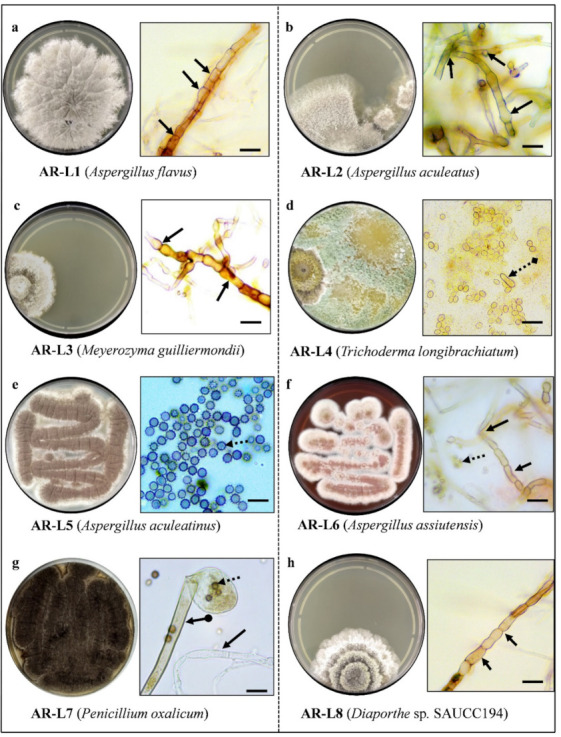
Pictorial representation of isolated fungal colony on PDA plate after 7 days of incubation and their microscopic view of fungal endophytes (**a**) *A. flavus*, (**b**) *A. aculeatus*, (**c**) *M. guilliermondii*, (**d**) *T. longibrachiatum*, (**e**) *A. aculeatinus*, (**f**) *A. assiutensis*, (**g**) *P. oxalicum* and (**h**) *Diaporthe* sp. SAUCC194 at 100× under bright field microscope. Mycelial septa (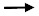), Spores (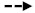), Conidia (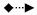) Conidiophore (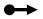).

From the leaf segment, eight fungal isolates were obtained by culture dependent method. The isolated fungal strains were designated as AR-L1 to AR-L8, for example; AR-L1 indicates the first fungal strain associated with the leaf of *A. rohituka*. Morphological characteristics of fungal isolates were observed under a microscope, and were identified based on their key morphological characteristics like shape and pigmentation of colony, morphology of mycelium (hyphae), presence/absence of septa and shape of conidia/spores (Figure 1). The fungal strains AR-L1, AR-L2, AR-L5 and AR-L6 showed similar characteristics as of *Aspergillus* sp. The characteristics found can be defined as powdery masses of whitish cream to black spores, septate and hyaline hyphae with conidia splitting into columns imparting rough texture to colonies were seen. Globose to sub-globose conidia and uniseriate vesicles were observed, along with the thick mycelial mat underneath the colonies [30]. The morphological characteristics of AR-L3 was found to be identical with *Meyerozyma guilliermondii* with features showing glabrous, white to cream-colored colonies. The identifiable character of AR-L4 was like *Trichoderma* sp. that possess conidiophores in small cottony pustules, and it forms in the scant aerial mycelium. Conidia was smooth, and the shape of conidia ranged from ellipsoidal to oblong or tuberculate to roughened or subglobose. AR-L7 was identified as of *Penicillium* sp. based on the identical character as a compact, powdery green-colored colony of which the back side appeared to be yellowish cream in color on a PDA plate. Mycelia was colorless with an arrangement of spores similar to a broom [31]. AR-L8 was identified as of *Diaporthe* sp. due to the presence of white, reverse off-white grey olivaceous coralloid, adpressed colonies and with no aerial mycelium [32].

### 3.2. Eight Fungal Endophyte Species Belonging to the Division Ascomycota were Identified

After sequencing, the BLAST search was performed for obtained raw sequences for ITS region. The species level identification of isolated fungal strains along with their complete 5.8 s ribosomal RNA gene sequences was done, and strains were identified as *Aspergillus flavus*, *Aspergillus aculeatus*, *Meyerozyma guilliermondii*, *Trichoderma longibrachiatum*, *Aspergillus aculeatinus*, *Aspergillus assiutensis*, *Penicillium oxalicum* and *Diaporthe* sp. SAUCC194 (named as AR-L1 to AR-L8, respectively) (Table S1). In order to study the phylogenetic relationship between the fungal endophytes, a phylogenetic tree was constructed.

The constructed phylogenetic tree was clustered into four clades. Clade I consisted of one isolate that is *P. oxalicum* (MK332586), whereas Clade II consisted of *Diaporthe* sp. SAUCC194 (MT822596), and Clade III consisted of three species, i.e., *A. aculeatinus* (MK281555) and *T. longibrachiatum* (MT634694) along with *M. guilliermondii* (MT598067), which belong to the sub clade of Clade III. Clade IV’s branch of the phylogenetic tree consisted of two closely related species, i.e., *A. flavus* (KX253948) and *A. aculeatus* (MT541887), whereas one more species belonged to the sub clade of Clade IV, i.e., *A. assiutensis* (MT640286) (Figure S2). In the current study, being the genera of *Aspergillus*, *A. aculeatinus* does not belong to Clade IV, for such a contrasting result, the differences in the season and geographical location of the plant could be one of the possible reasons.

### 3.3. EA Extract of P. oxalicum Contains Highest Amount of Phenolic and Flavonoid Content

Phenolic and flavonoid compounds and their derivatives have been considered as primary free radical scavenging molecules by virtue of their aromatic rings that contribute to their antioxidant properties. The result for total flavonoid content has shown a variation of approximately 9-fold ranging from 17.70 µg QE/mg to 155.69 µg QE/mg of EA extract isolated fungal endophytes (Table 1). The highest flavonoid content has been displayed by EA extract of *P. oxalicum* with 155.69 µg QE/mg of EA extract. The lowest flavonoid content has been observed for *A. flavus* showing value of 17.70 µg QE/mg of EA extract. A linear relationship has been obtained by plotting concentration of Quercetin and its absorbance at 420 nm (R^2^ = 0.9977) (Figure S3).

Total phenolic content produced by all the identified fungal endophytes revealed a variation of approximately 5-folds ranging from 23.40 µg GAE/mg to 120.99 µg GAE/mg of EA extract of isolated fungal endophytes (Table 1). The highest phenolic content value being 120.99 µg GAE/mg of EA extract for *P. oxalicum*, whereas the lowest phenolic content was observed for *M. guilliermondii* with of 23.40 µg GAE/mg of EA extract. The regression equation obtained from the calibration curve of gallic acid (R^2^ = 0.9891) has been used to calculate the total content of phenolic compounds and expressed as µg GAE/mg of EA extract (Figure S4).

The phenolic compounds have been reported to prevent oxidative damages in biological systems through oxygen scavenging, free radical inhibition, metal inactivation and peroxide decomposition [33]. Among different species of fungal endophytes, the alteration in phenolic and flavonoid profiles directly affects their biological activities such as antioxidant and cytotoxic activities. The highest phenolic content of 40.30 mg GAE/g of plant dry weight in fungal extract of *Talaromyces* sp. derived from two varieties of Egyptian artichoke, namely French Hyrious and Egyptian Baladi, has evidenced the highest total antioxidant capacity (681 mg ascorbic acid equivalent per gram dry weight) [34]. Another report showed that the contents of phenolic (204 ± 6.144, 312.3 ± 2.147 and 152.7 ± 4.958 µg GAE/mg of dry extract) and flavonoid (177.9 ± 2.911, 644.1 ± 4.202 and 96.38 ± 3.851µg RE/mg of dry extract) compounds of fungal strains *Alternaria alternata, Cladosporium cladosporioides* and *Alternaria brassicae* exhibited dose dependent radical scavenging activity [35]. The significant correlation of TPC and TFC with antioxidant activity has been supported through another study showing a fungal endophyte, *Nigrospora sphaerica* isolated from *Catharanthus roseus*, representing the highest TPC (0.030 ± 0.000 (mg GAE/g) and TFC (0.038 ± 0.001 (mg QE/g) with the highest reducing power activity [36]. Similarly, our findings are also in agreement with the fact that the presence of the phenolic and flavonoid content correlates with antioxidant and cytotoxic property, further suggesting that EA extract of *P. oxalicum* can serve as a promising candidate against various diseases caused by oxidative damage.

### 3.4. HPTLC Fingerprint Analysis Showed the Presence of Unique Bioactive Components in P. oxalicum Derived Bioactive Compounds

**Figure 2:**
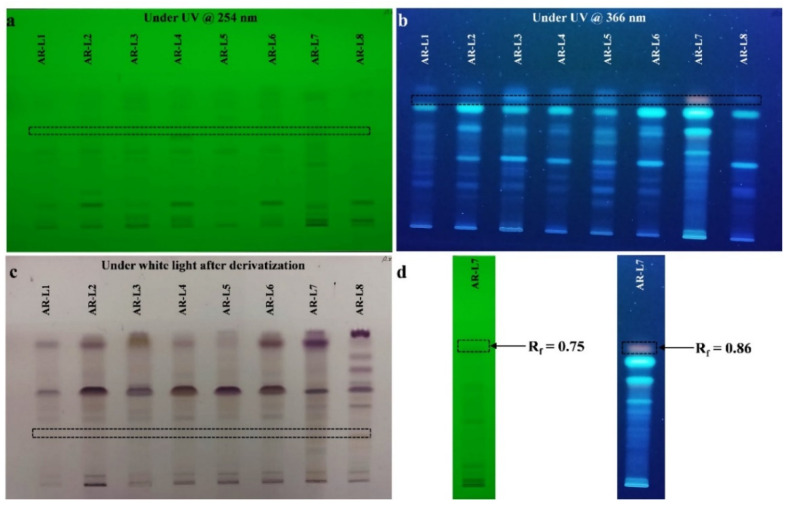
HPTLC fingerprint profiling of EA extract of fungal endophytes. (**a**) Image of TLC plate at 254 nm, (**b**) image of TLC plate at 366 nm, (**c**) image of TLC plate under white light after derivatization with anisaldehyde sulphuric acid reagent and (**d**) HPTLC fingerprint profiling of EA extract of *P. oxalicum* showing unique bioactive component with respective value of retention factor (R_f_).

The HPTLC fingerprinting analysis of EA extract of all identified fungal strains was carried out for the presence of bioactive compounds. Various combinations of polar and non-polar solvents were used for HPTLC separation, but the best separation of fungal extracts was obtained with a mobile phase of toluene: chloroform: ethyl alcohol (4:4:1, *v*/*v*/*v*). Image of the plate was captured at short UV range 254 nm and long UV range 366 nm, and band for bioactive components of each fungal extracts were clearly seen in chromatogram (Figure 2). HPTLC fingerprint scanned at 254 nm (D2 light source) revealed the presence of six different components in EA extract of *A. flavus*, *M. guilliermondii*, *T. longibrachiatum* and *A. assiutensis*. Scanning of EA of *A. aculeatus* and *A. aculeatinus* revealed the presence of 7 different components in each isolate. EA extract of *Diaporthe* sp. SAUCC194 showed 5 different components according to their R_f_ values and area percentage. EA extract of *P. oxalicum* showed 7 different major components, out of the seven components, one novel component is found with R_f_ value of 0.75 and area percentage value of 6.46%.

HPTLC fingerprint scanned at 366 nm (Hg lamp, fluorescence mode) for all the EA extract of fungal strains. EA extract of *A. flavus*, *A. aculeatus*, *M. guilliermondii*, *T. longibrachiatum* and *Diaporthe* sp. SAUCC194 showed four different major components. Similarly, *A. aculeatinus* possess seven different components, whereas in *A. assiutensis* and *P. oxalicum*, three different components are found. Out of three different bioactive components found in *P. oxalicum*, one unique band is observed with area percentage and R_f_ value of 62.50% and 0.74, respectively. Post-chromatography derivatization by anisaldehyde sulphuric acid reagent revealed the chemical nature of bioactive components such as terpenoids, steroids, sterols and saponins present in fungal extract. Out of eight EA extracts of isolated strains, the EA extract of *P. oxalicum* showed a unique band (a prominent violet color band indicates phenolic molecules [37]) that could be potential source of antioxidant and cytotoxic activity.

### 3.5. EA Extract of A. flavus and P. oxalicum Shows Potential Free Radical Scavenging Activity

**Figure 3:**
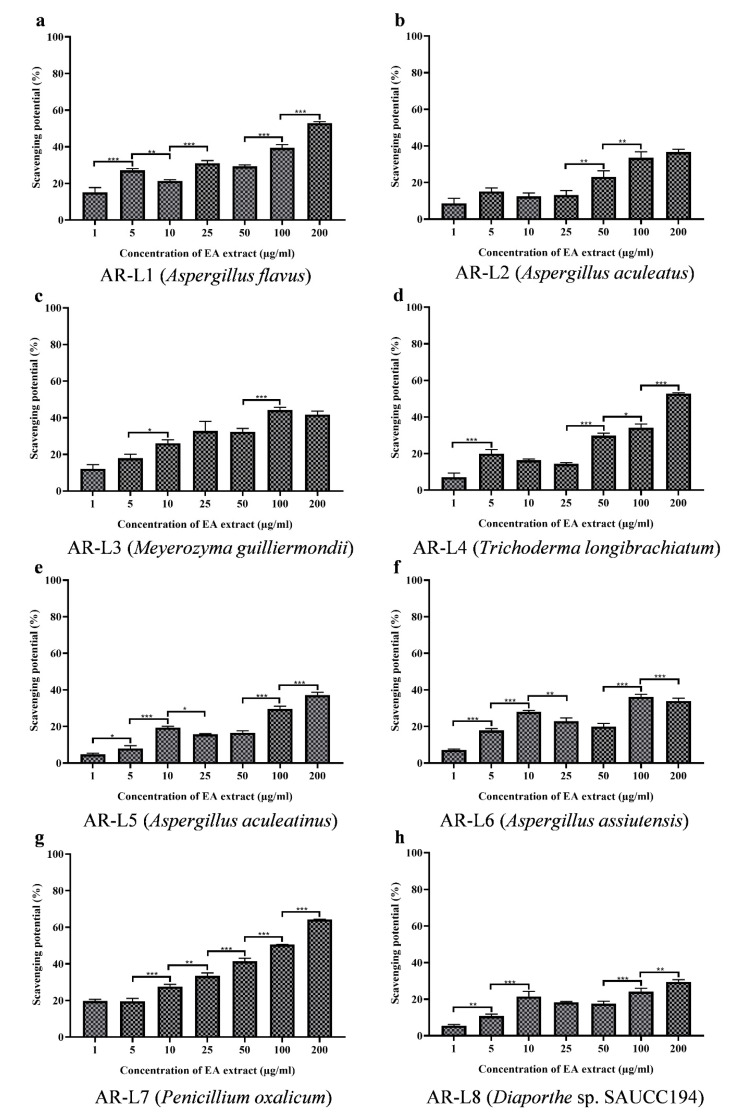
DPPH free radical scavenging potential of EA extract of fungal endophytes (**a**) *A. flavus*, (**b**) *A. aculeatus*, (**c**) *M. guilliermondii*, (**d**) *T. longibrachiatum*, (**e**) *A. aculeatinus*, (**f**) *A. assiutensis*, (**g**) *P. oxalicum* and (**h**) *Diaporthe* sp. SAUCC194. All the experiments were performed in triplicate. *p*-value was calculated by comparing means ± SD of the DPPH free radical scavenging potential (%), using one-way ANOVA followed by Tukey to determine statistical significance which are as follows; *** *p* ≤ 0.001; ** *p* ≤ 0.002; * *p* ≤ 0.033.

The antioxidant activity was observed visibly through the decolorization of purple color to yellow, while the percentage inhibition was estimated through spectrophotometric analysis at 517 nm. The antioxidant activity assessed for the fungal strains displayed that some of the strains possess significant antioxidant activity and rest other strains showed less or minimum antioxidant activity. Out of eight fungal isolates *P. oxalicum* showed best antioxidant activity as compared to other strains and in a concentration dependent manner (Figure 3). The EC_50_ value for *P. oxalicum* was found to be 96.98 ± 0.270 µg/mL. Another strain with significant antioxidant activity was EA extract of *A. flavus* that displayed moderate antioxidant activity with EC_50_ value of 178.30 ± 1.446 µg/mL. The other strains were least effective against free radicals and EC_50_ value calculated was out of tested range (>200 µg/mL). The positive control used was ascorbic acid that was used to compare the antioxidant potential of fungal endophyte derived bioactive compounds. The EC_50_ value of ascorbic acid (positive control) was estimated to be 10.60 ± 0.257 µg/mL (Table 2).

Results showed that the content of phenolic compounds was lower compared to the EA extract of *P. oxalicum*, but the antioxidant activity was found to be best among all strains. DPPH scavenging activity of EA extract of *A. flavus* was not in accordance to phenolic content and that indicated the involvement of other class of compounds responsible for displaying antioxidant activity. The method of estimation of phenolic content or interference of other compounds could be a possible reason for nonsignificant phenolic content produced by endophyte *A. flavus*. Other factors include the synergistic action of other class of compounds that showed potential antioxidant activity [38,39,40,41]. Some previous studies have also shown inconsistent correlation between phenolic content and antioxidant activity [42,43]. However, *A. flavus* have been reported with significant antioxidant activity and with a positive correlation with the phenolic content. The DPPH free radical scavenging activity of EA extract of *P. oxalicum* was in accordance with previous study, which showed that fungal endophyte *P. oxalicum* isolated from *Citrus limon* showed linear correlation between high phenolic content and DPPH scavenging activity with an EC_50_ value of 127.56 μg/mL [44]. The metabolites N-[2-(4 hydroxyphenyl) ethenyl] formamide and tuckolide isolated from *P. oxalicum* were effective against DPPH radical scavenging assay with EC_50_ values of 18.53 and 79.17 μg/mL, respectively [45].

### 3.6. EA Extract of A. flavus and P. oxalicum Display Potential Superoxide Anion Scavenging Activity

**Figure 4:**
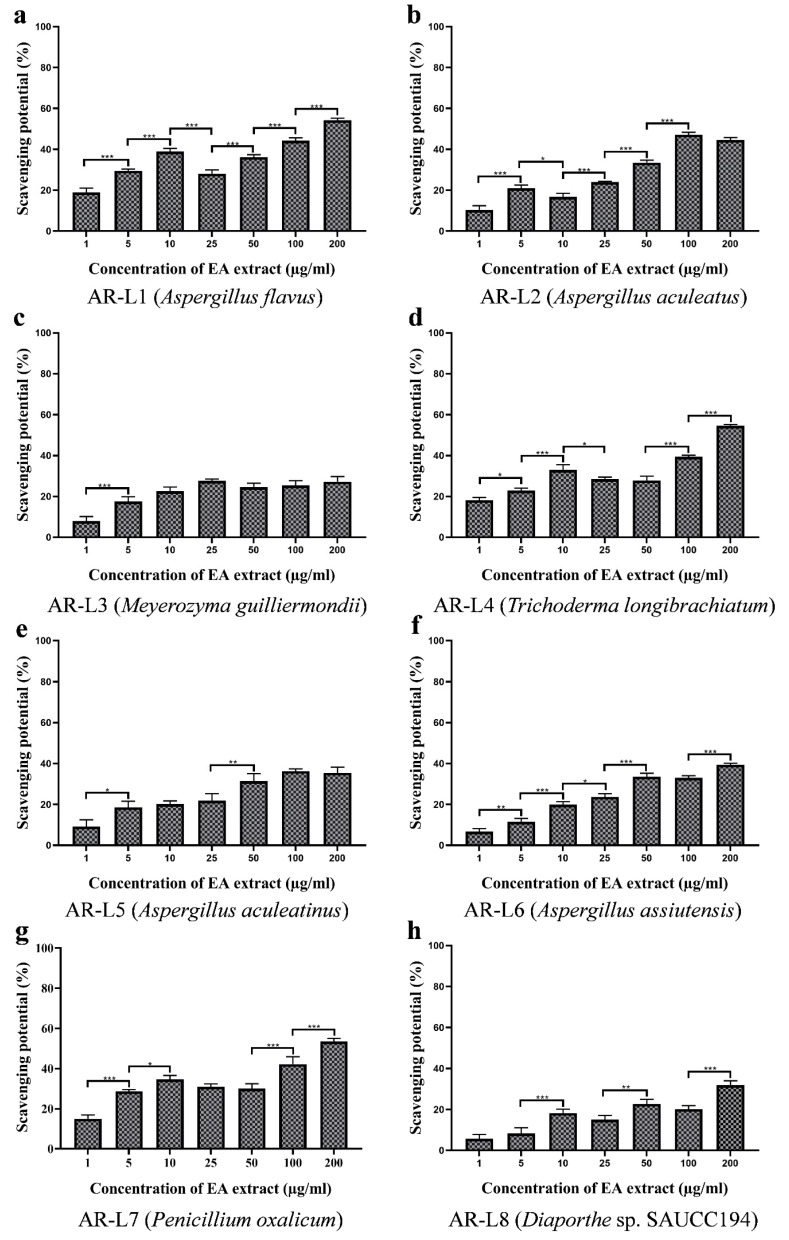
Superoxide anion scavenging potential of EA extract of fungal endophytes (**a**) *A. flavus*, (**b**) *A. aculeatus*, (**c**) *M. guilliermondii*, (**d**) *T. longibrachiatum*, (**e**) *A. aculeatinus*, (**f**) *A. assiutensis*, (**g**) *P. oxalicum* and (**h**) *Diaporthe* sp. SAUCC194. All the experiments were performed in triplicate. *p*-value was calculated by comparing means ± SD of the superoxide anion radical scavenging potential (%), using one-way ANOVA followed by Tukey to determine statistical significance, which are as follows; *** *p* ≤ 0.001; ** *p* ≤ 0.002; * *p* ≤ 0.033.

The superoxide radical scavenging assay performed for the eight strains showed significant inhibition of superoxide radicals. The inhibition percentage of super oxide anion radicals surged with the increasing concentration of fungal EA extract (Figure 4). Among eight fungal strains, the highest scavenging was observed for EA extract of *A. flavus* with an EC_50_ value of 157.52 ± 1.118 µg/mL, whereas the EA extract of *P. oxalicum* and *T. longibrachiatum* also exhibited a closer superoxide anion scavenging activity to EA extract of *A. flavus* with EC_50_ values of 169.28 ± 0.402 µg/mL and 170.43 ± 1.405 µg/mL, respectively. EA extracts of other five fungal endophytes, such as *A. aculeatus*, *M. guilliermondii*, *A. aculeatinus*, *A. assiutensis* and *Diaporthe* sp. SAUCC194 showed no superoxide anion scavenging activity within tested range of concentration, and therefore may exhibit an EC_50_ value of more than 200 µg/mL (Table 2). The standard oxidant or positive control (Ascorbic acid) exhibited EC_50_ value of 33.36 ± 1.186 µg/mL. All the fungal extracts showed lower antioxidant activity than the positive control.

The superoxide radicals are the most commonly formed radicals in the biological system through various enzymatic, non-enzymatic and autooxidation reactions and cause several disorders and pathological effects [46]. The results observed do not show linear correlation between phenolic content and antioxidant activity. Previous reports have witnessed the role of exopolysaccharide As1-1 produced by the mangrove endophytic fungus *Aspergillus* sp. Y16 in the scavenging of superoxide radicals with an EC_50_ value of 3.4 mg/mL [47]. These reports have shown that *Aspergillus* sp. exhibit superoxide anion scavenging and could be a potential natural antioxidant. The moderate superoxide anion scavenging activity of *P. oxalicum* can be related to its high phenolic content. In a report, the fungal endophyte *P. oxalicum* isolated from *Ligusticum chuanxiong* Hort (CX) was reported to exhibit superoxide anion scavenging activity with an IC_50_ value of 1.41 ± 0.22 mg/mL and contain high polyphenolic content [48]. EA extract of *T. longibrachiatum* showed moderate antioxidant activity. The previous report has shown mangrove-derived *Trichoderma* sp. to possess significant superoxide anion scavenging activity of 71.45 % at 500 μg/mL. However, the phenolic content was not reported to be significant for *T. longibrachiatum.* Besides, other strains with phenolic content did not exhibit significant antioxidant activity, and EC_50_ value was observed to be >200 µg/mL.

### 3.7. EA Extract of T. longibrachiatum and P. oxalicum Exhibit Significant Hydroxyl Radical Scavenging Activity

**Figure 5:**
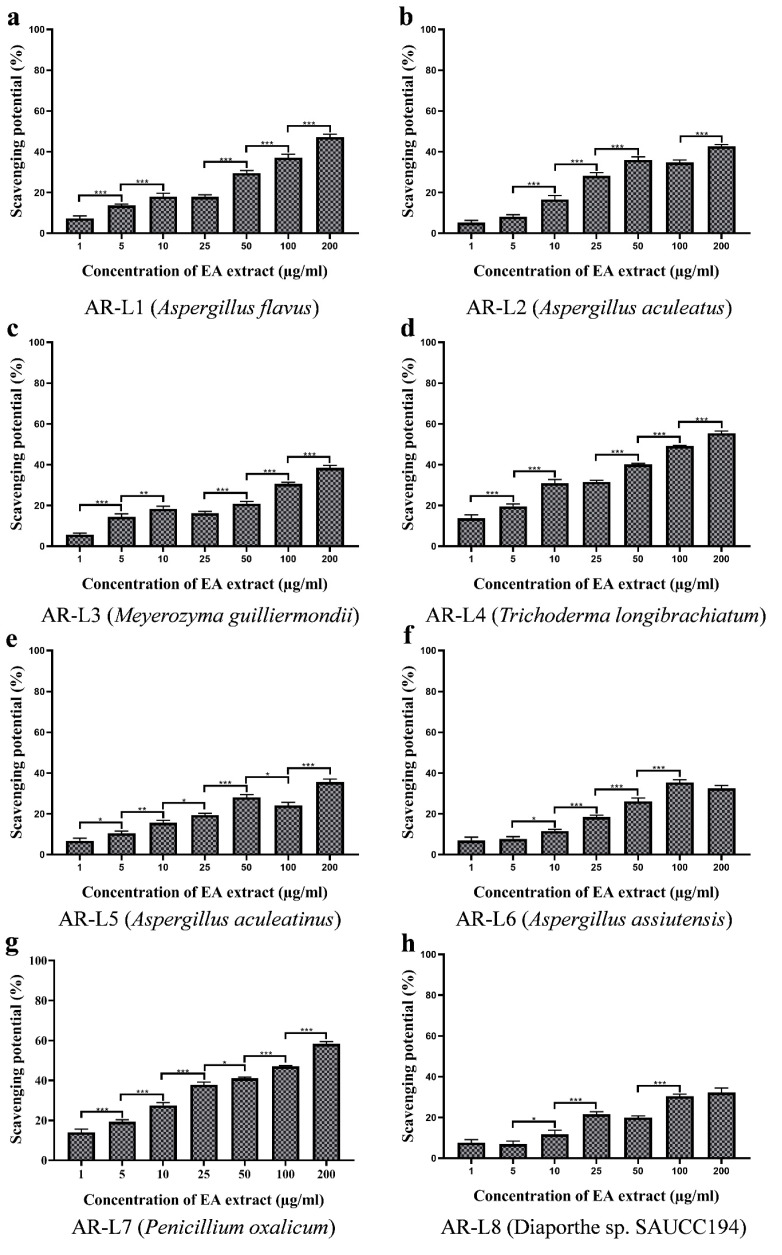
Hydroxyl radical scavenging potential of EA extract of fungal endophytes (**a**) *A. flavus*, (**b**) *A. aculeatus*, (**c**) *M. guilliermondii*, (**d**) *T. longibrachiatum*, (**e**) *A. aculeatinus*, (**f**) *A. assiutensis*, (**g**) *P. oxalicum* and (**h**) *Diaporthe* sp. SAUCC194. All the experiments were performed in triplicate. *p*-value was calculated by comparing means ± SD of the hydroxyl radical scavenging potential (%), using one-way ANOVA followed by Tukey to determine statistical significance, which are as follows; *** *p* ≤ 0.001; ** *p* ≤ 0.002; * *p* ≤ 0.033.

The hydroxyl radical scavenging activity was evaluated on the basis of percentage inhibition of deoxyribose degradation. The formation of pink chromogen was measured at 535 nm. The EA extract of fungal isolates showed concentration-dependent inhibition of hydroxyl radicals (Figure 5). The highest EC_50_ value was observed to be 113.30 ± 1.206 µg/mL for EA extract of *T. longibrachiatum*, which showed significant hydroxyl radicals scavenging activity, whereas EA extract of *P. oxalicum* exhibited moderate hydroxyl radical scavenging activity with an EC_50_ value of 126.12 ± 0.636 µg/mL. Beside the aforementioned strains, EA extracts of mycelia of rest other strains isolated from leaf and twig showed minimum antioxidant activity with EC_50_ value of more than 200 µg/mL (Table 2). The EC_50_ value for ascorbic acid used as positive control was reported to be 24.37 ± 1.116 µg/mL.

The strongest oxidizing power is known for hydroxyl anion, as it non-specifically targets protein, lipid and nucleic acids and leads to the development of serious diseases. The hydroxyl anion scavenging assay colorimetrically measures the formation of colored product malondialdehyde. The EA extract of *T. longibrachiatum* prevented the reaction in a concentration-dependent manner, and maximum antioxidant activity was recorded with an EC_50_ value of 113.30 ± 1.206 µg/mL, but it was not linearly correlated to its phenolic content. The linear correlation between phenolic content and hydroxyl anion scavenging has been observed for *Trichoderma* strain EMFCAS8, a mangrove endophytic fungus [49]. The considerable hydroxyl anion scavenging activity of EA extract of *P. oxalicum* has been validated through higher phenolic content. In a report, *P. oxalicum* isolated from *Citrus limon* has shown concentration dependent scavenging of hydroxyl radical that was attributed to its polyphenolic contents [43]. The fungal endophyte *P. oxalicum* isolated from *Ligusticum chuanxiong* Hort (CX) showed strong correlation between polyphenolic content and hydroxyl anion scavenging and EC_50_ value for scavenging activity was observed to be 3.38 ± 0.04 mg/mL [48]. These results suggest that the fungal endophytes *P. oxalicum* and *T. longibrachiatum* isolated from *A. rohituka* could serve as a potential candidate against oxidative damage.

### 3.8. EA Extract of T. longibrachiatum and P. oxalicum Shows Maximum Nitric Oxide Scavenging Activity

**Figure 6:**
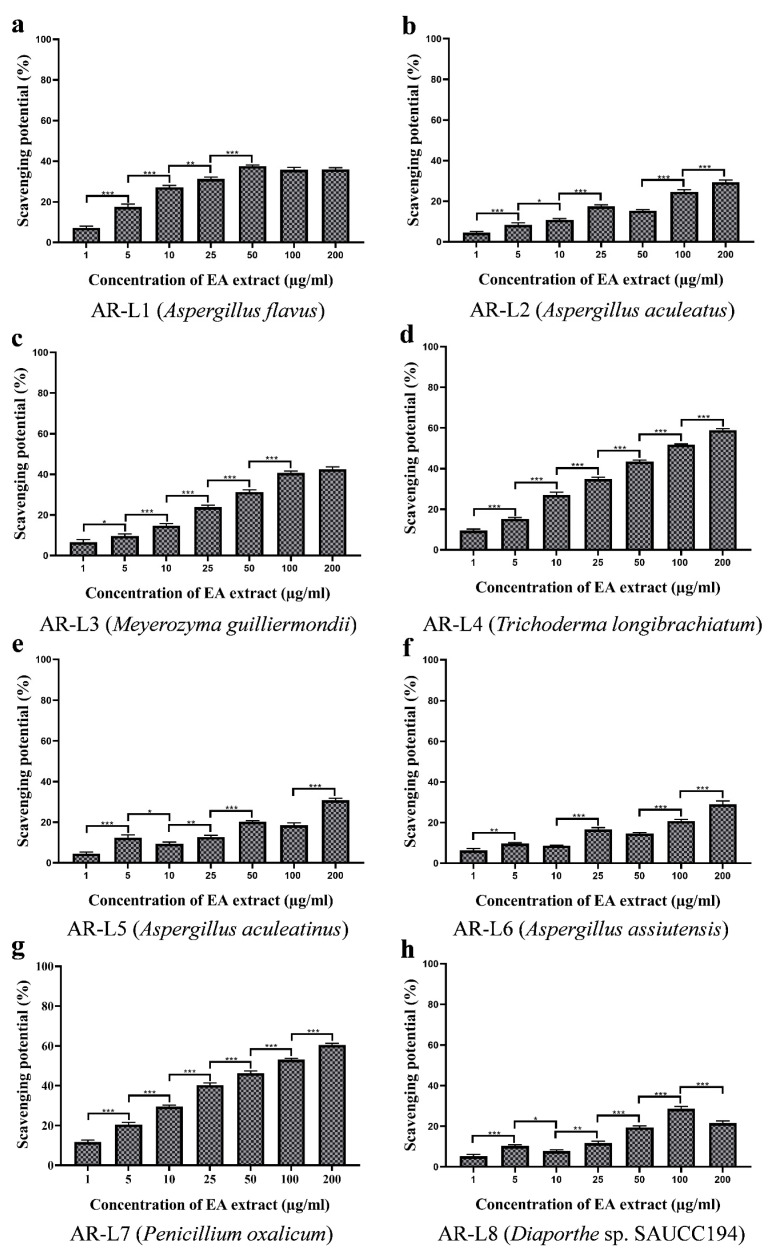
Nitric oxide scavenging potential of EA extract of fungal endophytes (**a**) *A. flavus*, (**b**) *A. aculeatus*, (**c**) *M. guilliermondii*, (**d**) *T. longibrachiatum*, (**e**) *A. aculeatinus*, (**f**) *A. assiutensis*, (**g**) *P. oxalicum* and (**h**) *Diaporthe* sp. SAUCC194. All the experiments were performed in triplicate. *p*-value was calculated by comparing means ± SD of the nitric oxide radical scavenging potential (%), using one-way ANOVA followed by Tukey to determine statistical significance, which are as follows; *** *p* ≤ 0.001; ** *p* ≤ 0.002; * *p* ≤ 0.033.

The chain of reactions leading to generation of nitrite ions was arrested through the EA extract of fungal isolates. The nitric oxide scavenging activity of EA extract was observed to be increased through the increasing concentration of extract (Figure 6). The significant antioxidant was observed for the fungal EA extracts and was comparable to the activity shown by positive control. The strongest activity was shown by the fungal isolate *P. oxalicum* with an observed EC_50_ value of 75.79 ± 0.692 µg/mL. Another strain to show significant scavenging of nitric oxide radical was *T. longibrachiatum* with an EC_50_ value of 89.14 ± 0.894 µg/mL EC_50_ value of ascorbic acid (positive control) was estimated to be 21.75 ± 0.566 µg/mL. EA extract of *A. flavus*, *A. aculeatus*, *M. guilliermondii*, *A. aculeatinus*, *A. assiutensis*, and *Diaporthe* sp. SAUCC194 showed minimum antioxidative activity with EC_50_ value of more than 200 µg/mL (Table 2).

The reactive oxygen species generated through various metabolic process pose deleterious effects to specific biomolecules such as lipid and proteins [50]. The presence of unpaired electron in nitric oxide causes it to act as a free radical and damage biomolecules on interaction. Natural antioxidants like endophytic fungal extract are known to be safe and bioactive for neutralizing the negative effects of free radicals. The nitric oxide scavenging assay revealed the fungal endophyte *P. oxalicum* to show highest antioxidant activity among other isolates, which is attributed to the increased phenolic and flavonoid contents. The fungal strain *P. oxalicum* derived from marine sources has been observed to exhibit strong inhibitory activities for NO production with 57.6% [51]. Another strain, *T. longibrachiatum*, showed moderate antioxidant activity with EC_50_ value of 89.14 ± 0.894 µg/mL that can be related to its moderate phenolic content. Previous report has revealed significant NO_2_ radical scavenging activity of mangrove derived fungal endophyte *Trichoderma* strain EMFCAS8 that was highly correlated with phenolic content [49]. Overall, results of antioxidant activity also show that EA extract of endophytes *A. flavus*, *A. aculeatus*, *M. guilliermondii*, *A. aculeatinus*, *A. assiutensis*, and *Diaporthe* sp. SAUCC194 exhibited minimum scavenging potential against tested synthetically generated free radicals having a certain extent of phenolic and flavonoid content. This is may be due to the antagonistic effect of phenolic and flavonoid compounds produced by fungal endophytes [52,53].

### 3.9. EA Extract of P. oxalicum Showed Promising Cytotoxic Activity against Cancer Cells

**Figure 7:**
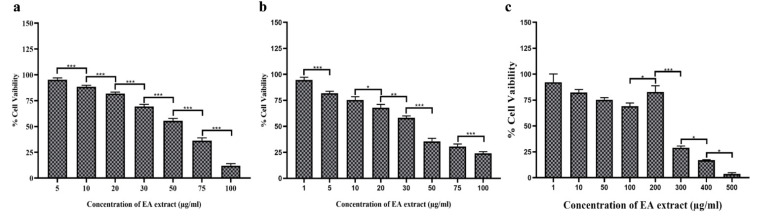
(**a**) Cytotoxic effect of EA extract of *P. oxalicum* against HuT-78 cell line (Human T cell lymphoma cell line), (**b**) MDA-MB-231 (breast cancer cell line) and (**c**) MCF-7 cell line (Human breast cancer cell line). All experiments were performed in triplicate. *p*-value was calculated by comparing means ± SD of percentage of cell viability of cancer cell, using one-way ANOVA followed by Tukey to determine statistical significance, which are as follows; *** *p* ≤ 0.001; ** *p* ≤ 0.002; * *p* ≤ 0.033.

Among eight identified endophytes, EA extract of *P. oxalicum* exhibited potential antioxidant activity, increased contents of phenolic and flavonoid compounds and produces more and unique bioactive components therefore, further selected to evaluate the cytotoxic activity against three different human cancer cell lines (HuT-78, MDA-MB-231 and MCF-7) (Figure 7a–c). Our result showed that EA extract of *P. oxalicum* exhibited cytotoxic activity against breast cancer and T lymphoma cells in a dose dependent manner. The IC_50_ values for EA extract of *P. oxalicum* were observed to be 56.81 ± 0.671 µg/mL for HuT-78, 37.24 ± 1.26 µg/mL for MDA-MB-231 and 260.62 ± 5.415 µg/mL for MCF-7 cells (Table 3). Taken together, our findings suggest that the EA extract of *P. oxalicum* has potential anticancer activity and thus might be considered as a therapeutic option for cancer treatment. Cancer is caused due to the development of abnormal cells that grow beyond their limits and through metastasis invade other organs of the body and pose damages. The proliferation of cells leading to cancer is significantly inhibited by fungal endophytes derived bioactive compounds. Previous studies have shown anticancer potential of fungal endophyte isolated from *Datura stramonium* against UMG87 glioblastoma and A549 lung carcinoma cell lines using MTT assay [54]. In another report, a bioactive compound flavipin isolated from an endophytic fungus associated with the *Chaetomium globosum* inhibited the growth of A549_,_ MCF-7, and HT-29 cancer cell line with an IC_50_ value of 9.549 µg/mL, 54 µg/mL and 18 µg/mL, respectively [55]. In a study, it was found that a compound ergoflavin isolated from an endophytic fungus *P. oxalicum* caused cytotoxicity in HCT116, Panc1, ACHN and H460 cancer cell line with IC_50_ values of 8.0 ± 0.45 µM, 2.4 ± 0.02 µM, 1.2 ± 0.20 µM and 4.0 ± 0.08 µM, respectively [56]. In one of the recent reports, it was found that a secondary metabolite obtained from marine endophytic fungi *P. chrysogenum* isolated from green algae showed anticancer activity against the MCF-7 cancer cell line with an IC_50_ value of 101.1 µg/mL [57].

### 3.10. Comparative Analysis between EA Extract of AR-L7 and P. oxalicum (PO-01) Isolated from Rhizospheric Soil of Maize

**Figure 8:**
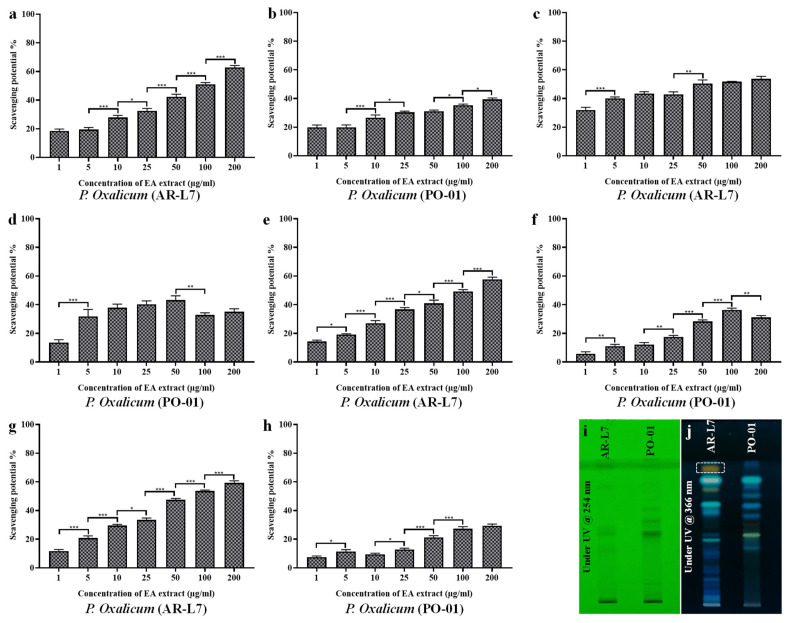
Comparative evaluation of antioxidant potential (**a**,**b**) DPPH free radical scavenging, (**c**,**d**) superoxide anion scavenging, (**e**,**f**) hydroxyl radical scavenging, and (**g**,**h**) nitric oxide scavenging for the EA extract of fungal strains *P. Oxalicum* (AR-L7) isolated from *A. rohituka* and *P. Oxalicum* (PO-01) isolated from rhizospheric soil of maize. All the experiments were performed in triplicate. *p*-value was calculated by comparing means ± SD of the free radical scavenging potential (%), using one-way ANOVA followed by Tukey to determine statistical significance. Statistical significances are as follows; *** *p* ≤ 0.001; ** *p* ≤ 0.002; * *p* ≤ 0.033. HPTLC fingerprint profiling of EA extract of fungal endophytes *P. Oxalicum* (AR-L7) and *P. Oxalicum* (PO-01) (**i**) image of TLC plate at 254 nm, (**j**) image of TLC plate at 366 nm.

Results of phenolic and flavonoid contents, antioxidant and cytotoxic activity of EA extract of fungal endophyte *P. oxalicum* have compelled us to perform a host-dependent activity and biochemical profiling of *P. oxalicum* isolated from two different hosts. In the present study, we have compared the antioxidant activity of EA extract of fungal endophyte *P. oxalicum* associated with leaf of *A. rohituka* (AR-L7) and rhizospheric soil of maize (PO-01). The results showed the fungal strain AR-L7 exhibit significant scavenging potential against all the synthetically generated oxidative radicals such as DPPH free radicals, super oxide anion radicals, hydroxyl radicals and nitric oxide radicals. The EC_50_ value of EA extract of AR-L7 was found to be 83.76 ± 1.14 µg/mL, 162.12 ± 1.185 µg/mL, 123.62 ± 2.01 µg/mL and 75.60 ± 1.31 µg/mL, for DPPH radical scavenging (Figure 8a,b), superoxide anion scavenging (Figure 8c,d), hydroxyl radical scavenging (Figure 8e,f) and nitric oxide scavenging assay (Figure 8g,h), respectively. The fungal strain PO-01 isolated from rhizospheric soil of maize failed to scavenge 50% of oxidative radicals within tested range of concentration and therefore, possessed EC_50_ value more than 200 µg/mL (Table 4). The screening of bioactive components has been done through HPTLC fingerprinting that showed *P. oxalicum* derived from *A. rohituka* (AR-L7) to possess more bioactive components as compared to the similar strain isolated from rhizospheric soil of maize (PO-01). HPTLC fingerprint scanned at 254 nm (D2 light source) revealed the presence of six different major components in EA extract of AR-L7 whereas, in PO-01, there were five major components according to their R_f_ values and area percentage. HPTLC fingerprint scanned at 366 nm (Hg lamp, fluorescence mode) for the EA extract of AR-L7 showed three different major components (Figure 8i,j) whereas, PO-01 showed that four major components were present according to their R_f_ values and area percentage.

The comparative study revealed that fungal endophyte *P. oxalicum* isolated from *A. rohituka* produces unique bioactive components and shows potential antioxidant activity as compared to a similar strain isolated from rhizospheric soil of maize. Our result reveals the host-dependent activity of fungal endophyte *P. oxalicum*. The fungal endophyte and its host establish a flexible relationship whose directionality is driven by the host genotype. Host genotypes shape the colonization of fungal endophytes and also influence their biological activity. In addition, host recognition and response to the colonized fungal endophytes and fungal gene expressions determine the type of interaction that may be either positive, negative and neutral [58,59,60]. The activity of fungal endophytes in different hosts vary in response to ecological, physiological and genetic factors [61]. The biochemical characteristics of fungal endophytes is directed through their association with the host. The bioactive components through HPTLC fingerprinting revealed that in the EA extract of AR-L7 (*P. oxalicum* isolated from *A. rohituka*), six major bioactive components were present, whereas it revealed the presence of five major components in EA extract of PO-01 (*P. oxalicum* isolated from rhizospheric soil of maize). The association of fungal endophytes with the host drives the qualitative and quantitative synthesis of bioactive compounds, and therefore affects their biological activity. The significant antioxidant activity of AR-L7 may be attributed due to the presence of unique bioactive component observed through HPTLC fingerprint analysis. Previous studies have shown a toxic metabolite, Secalonic acid D derived from *P. oxalicum* cause acute toxicoses in rats, mice and ducklings and also showed toxicity to male Swiss albino rats [62]. In other reports, fungal endophyte *P. oxalicum* have been reported for the presence of cytotoxic compounds dihydrothiophene-condensed chromones [63], production of potential antibiotics against pathogenic fungal strains [64] and antibacterial and antitumor activities [65]. Therefore, it can be concluded that the diversity, distribution and biological activity of fungal endophytes is determined through their response against interaction with host and that is attributed to their genetic background, ecological and physiological factors.

## 4. Conclusions

The present study revealed eight fungal endophytes isolated from the medicinal plant *A. rohituka* and their bioactive compounds were assessed for various antioxidant and cytotoxic activity. The fungal endophyte, *P. oxalicum* has been identified to exhibit potential biological activity in terms of antioxidant activity by virtue of its significant flavonoid and phenolic content. The unique band obtained for *P. oxalicum* through HPTLC fingerprinting also validates the significant activity of this fungal endophyte. The cytotoxic assay performed for EA extract of *P. oxalicum* has showed effective and significant cytotoxicity against human breast cancer cells (MDA-MB-231). Host-dependent activity revealed that EA extract of fungal endophyte *P. oxalicum* isolated from the leaf of *A. rohituka* produces more bioactive components and, therefore, possessed significant antioxidant activity over *P. oxalicum* isolated from rhizospheric soil of maize. Conclusively, the present study further directs the characterization and identification of bioactive compounds present in the EA extract of *P. oxalicum* with antioxidant and anticancer activity, and further appeals for its future examination in the improvement of human health.

## Figures and Tables

**Table 1:** Total phenolic content and flavonoid content present in EA extract of identified fungal strains associated with the leaf of *A. rohituka*.

S. No.	Species Identified	Total Phenolic Content (µg GAE/mg of EA Extract)	Total Flavonoid Content (µg QE/mg of EA Extract)
1.	*A. flavus*	45.65	17.70
2.	*A. aculeatus*	33.68	34.16
3.	*M. guilliermondii*	23.40	60.08
4.	*T. longibrachiatum*	32.42	78.88
5.	*A. aculeatinus*	69.36	32.65
6.	*A. assiutensis*	72.19	83.40
7.	*P. oxalicum*	120.99	155.69
8.	*Diaporthe* sp. SAUCC194	78.91	55.14

**Table 2:** Antioxidant activity of EA extracts of identified fungal strains associated with the leaf of *A. rohituka*.

S. No.	Species	EC_50_ Value (µg/mL)			
		DPPH Free Radical Scavenging Assay	Superoxide Anion Scavenging Activity	Hydroxyl Radical Scavenging Assay	Nitric Oxide Scavenging Assay
1.	*A. flavus*	178.30 ± 1.446	157.52 ± 1.118	>200	>200
2.	*A. aculeatus*	>200	>200	>200	>200
3.	*M. guilliermondii*	>200	>200	>200	>200
4.	*T. longibrachiatum*	>200	170.43 ± 1.405	113.30 ± 1.206	89.14 ± 0.894
5.	*A. aculeatinus*	>200	>200	>200	>200
6.	*A. assiutensis*	>200	>200	>200	>200
7.	*P. oxalicum*	96.98 ± 0.270	169.28 ± 0.402	126.12 ± 0.636	75.79 ± 0.692
8.	*Diaporthe* sp. SAUCC194	>200	>200	>200	>200
Positive control	Ascorbic Acid	10.60 ± 0.257	33.36 ± 1.186	24.37 ± 1.116	21.75 ± 0.566

**Table 3:** Cytotoxic activity of EA extract of *P. oxalicum* associated with the leaf of *A. rohituka*.

**Isolated Strains**	**Species**	**IC_50_ Value (µg/mL)**		
		**T-Cell Lymphoma Cancer Cell** **(HuT-78)**	**Human Breast Cancer Cell** **(MDA-MB-231)**	**Human Breast Cancer Cell** **(MCF-7)**
AR-L7	*P. oxalicum*	56.81 ± 0.617	37.24 ± 1.26	260.627 ± 5.415

**Table 4:** Host-dependent antioxidant activity of EA extract of fungal endophyte *P. oxalicum* (AR-L7) and PO-01.

Fungal Strains	Host Plant	EC_50_ Value (µg/mL)			
		DPPH Free Radical Scavenging Assay	Superoxide Anion Scavenging Assay	Hydroxyl Radical Scavenging Assay	Nitric Oxide Scavenging Activity
AR-L7 (*P. oxalicum*)	Leaf of *A. rohituka*	>200	>200	>200	>200
PO-01 (*P. oxalicum*)	Rhizospheric soil of maize	83.760 ± 1.14 µg/mL	162.12 ± 1.185 µg/mL	123.62 ± 2.01 µg/mL	75.60 ± 1.31 µg/mL

## Data Availability

All the data are mentioned in the manuscript and as supplementary materials.

## Supplementary Materials

The following supporting information can be downloaded at: https://www.mdpi.com/article/10.3390/jof8030285/s1. Table S1. List of leaf associated fungal strains identified through DNA sequencing of ITS region and their accession number. Figure S1. Gel image of PCR products of conserved ITS region of fungal strains associated with the leaf of *Amoora rohituka.* Figure S2. Phylogenetic tree of fungal endophytes constructed by maximum likelihood bootstrap (MLBS) method. Figure S3. Calibration curve for Quercetin (Standard). Figure S4. Calibration curve for Gallic acid (Standard). Refs. [66,67,68,69,70] are cited in Table S1.
