# Supplementary material for: Assessment of Biological Activities of Fungal Endophytes Derived Bioactive Compounds Isolated from Amoora rohituka
Source: J Fungi (Basel). 2022 Mar 10;8(3):285. doi: 10.3390/jof8030285 (PMC8952687; doi:10.3390/jof8030285)
Supplement: Supplementary file 1 [file jof-08-00285-s001.zip › jof-1607204-supplementary.pdf]

Table S1. List of leaf associated fungal strains identified through DNA sequencing of ITS region and their accession number.

S. no.	Isolated strains	Primer	Primer Sequence	Name of Organism identified through BLAST	Accession number	Morphological characteristics	Reference
**1**	AR-L1	ITS4 & ITS5	5'-TCCTCCGCTTATTGATATGC-3' 5'-GGAAGTAAAAGTCGTAACAAGG-3'	*Aspergillus flavus*	*KX253948*	Powdery masses of yellowish-green spores, Hyphae are septate and hyaline, thread-like branching and produces mycelia. Conidia producing thick mycelial mats are often seen.	[1]
**2**	AR-L2	ITS4 & ITS5	5'-TCCTCCGCTTATTGATATGC-3' 5'-GGAAGTAAAAGTCGTAACAAGG-3'	*Aspergillus aculeatus*	*MT541887*	Spores are black in color. They had white mycelia that grew inconspicuously on the colonies giving it a greyish appearance. Beneath the colonies they formed a thick a mat of mycelia which were visible at the edges.	[2]
**3**	AR-L3	ITS4 & ITS5	5'-TCCTCCGCTTATTGATATGC-3' 5'-GGAAGTAAAAGTCGTAACAAGG-3'	*Meyerozyma guilliermondii*	*MT598067*	Colonies are flat, moist, smooth, and cream to yellow in color, produces clusters of small blastospores along the pseudohyphae. Spherical to subspherical budding yeast-like cells. White to cream-colored smooth, glabrous, yeast-like colonies.	[3]
**4**	AR-L4	ITS4 & ITS5	5'-TCCTCCGCTTATTGATATGC-3' 5'-GGAAGTAAAAGTCGTAACAAGG-3'	*Trichoderma longibrachiatum*	*MT634694*	Sporulating at ≥ 35°C; a diffusing yellow pigment, Conidiophores forming in the scant aerial mycelium and in small, cottony pustules. Conidia typically ellipsoidal to oblong, smooth, less frequently subglobose or roughened to tuberculate.	[4]
**5**	AR-L5	ITS4 & ITS5	5'-TCCTCCGCTTATTGATATGC-3' 5'-GGAAGTAAAAGTCGTAACAAGG-3'	*Aspergillus aculeatinus*	*MK281555*	Conidial heads radiate, splitting into poorly defined columns or 2–6 well-defined columns with age, stipes are short, walls thin, smooth, hyaline, vesicles globose uniseriate, phialides flask-shaped and cover the entire surface of the vesicle, conidia subglobose to ellipsoidal, echinulate.	[5]
**6**	AR-L6	ITS4 & ITS5	5'-TCCTCCGCTTATTGATATGC-3' 5'-GGAAGTAAAAGTCGTAACAAGG-3'	*Aspergillus assiutensis*	*MT640286*	Conidia are globose to subglobose, pyriform, up to 6 µm or more. Conidial heads are whitish-orange, after	[6]

						long incubation sclerotia are creamish in color, conidiogenous cells are uniseriate.	
**7**	AR-L7	ITS4 & ITS5	5’-TCCTCCGCTTATTGATATGC-3’ 5’-GGAAGTAAAAGTCGTAACAAGG-3’	*Penicillium oxalicum*	*MK332586*	Colony was dark green color, powdery, compact and the back side of colony was yellowish cream in color on PDA media with colorless mycelium and the conidia, spores arrangements resembled a broom.	[7]
**8**	AR-L8	ITS4 & ITS5	5’-TCCTCCGCTTATTGATATGC-3’ 5’-GGAAGTAAAAGTCGTAACAAGG-3’	*Diaporthe* sp. SAUCC194	*MT822596*	Presence of white, reverse off-white grey olivaceous coralloid, adpressed colonies covering the entire plate of PDA and with no aerial mycelium.	[8]

Figure legends:

Figure S1. Gel image of PCR products of conserved ITS region of fungal strains associated with the leaf of *Amoora rohituka.* **(L)** Marker, **(A)** AR-L1**, (B)** AR-L2**, (C)** AR-L3**, (D)** AR-L4**, (E)** AR-L5**, (F)** AR-L6**, (G)** AR-L7**, (H)** AR-L8**,**

Figure S2. Phylogenetic tree of fungal endophytes constructed by maximum likelihood bootstrap (MLBS) method. The sequences were aligned through MUSCLE alignment program and the evolutionary history was inferred by using the Maximum Likelihood bootstrap (MLBS) method and General Time Reversible model.

Figure S3. Calibration curve for Quercetin (Standard).

Figure S4. Calibration curve for Gallic acid (Standard).

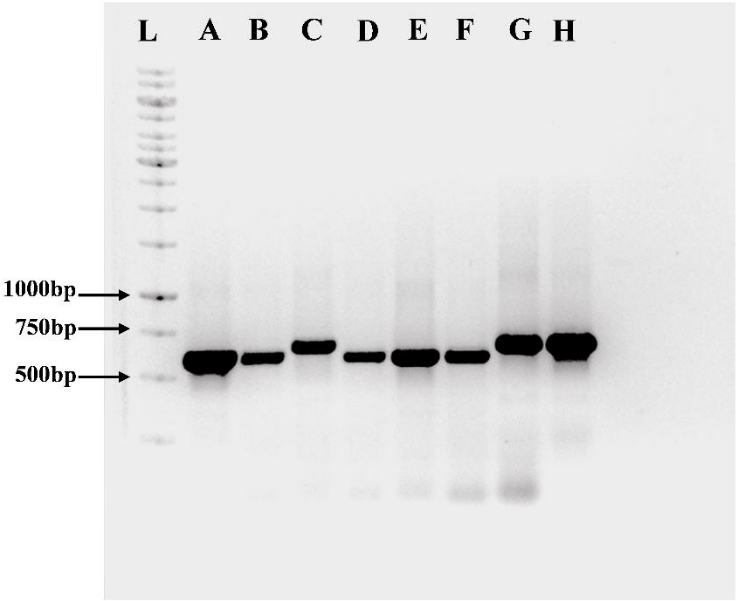

Figure S1. Gel image of PCR products of conserved ITS region of fungal strains associated with the leaf of *Amoora rohituka*. **(L)** 1Kb ladder, **(A)** AR-L1**, (B)** AR-L2**, (C)** AR-L3**, (D)** AR-L4**, (E)** AR-L5**, (F)** AR-L6**, (G)** AR-L7**, (H)** AR-L8**.**

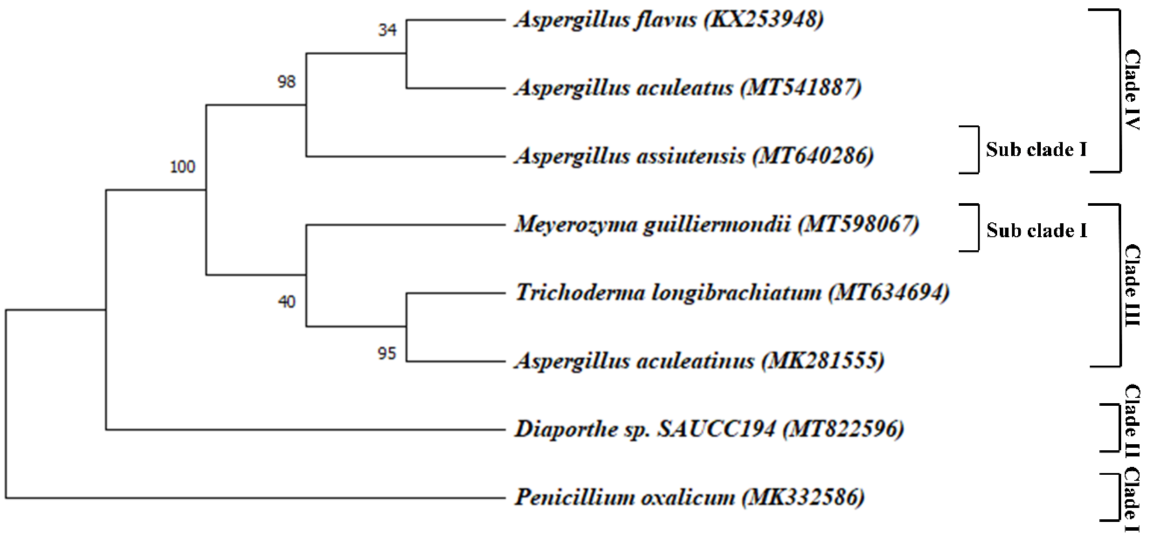

Figure S2. Phylogenetic tree of fungal endophytes constructed by maximum likelihood bootstrap (MLBS) method. The sequences were aligned through MUSCLE alignment program and the evolutionary history was inferred by using the Maximum Likelihood bootstrap (MLBS) method and General Time Reversible model.

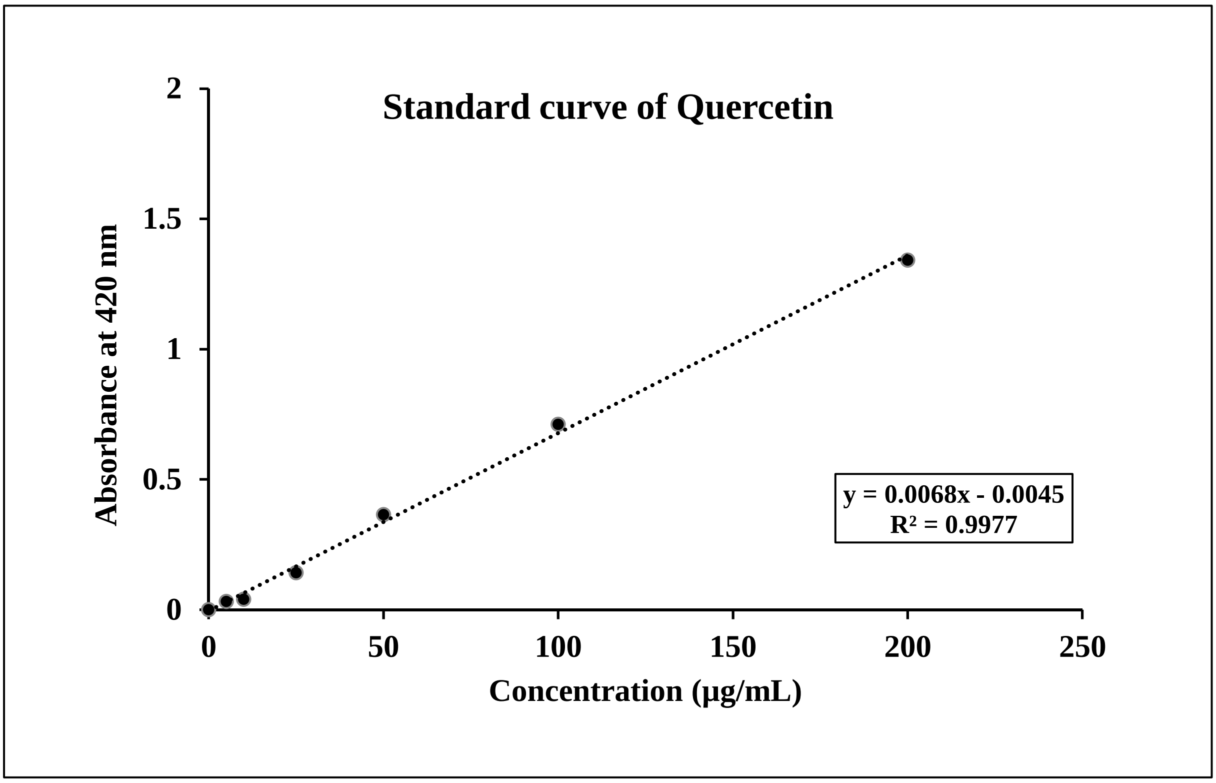

Figure S3. Calibration curve for Quercetin (Standard).

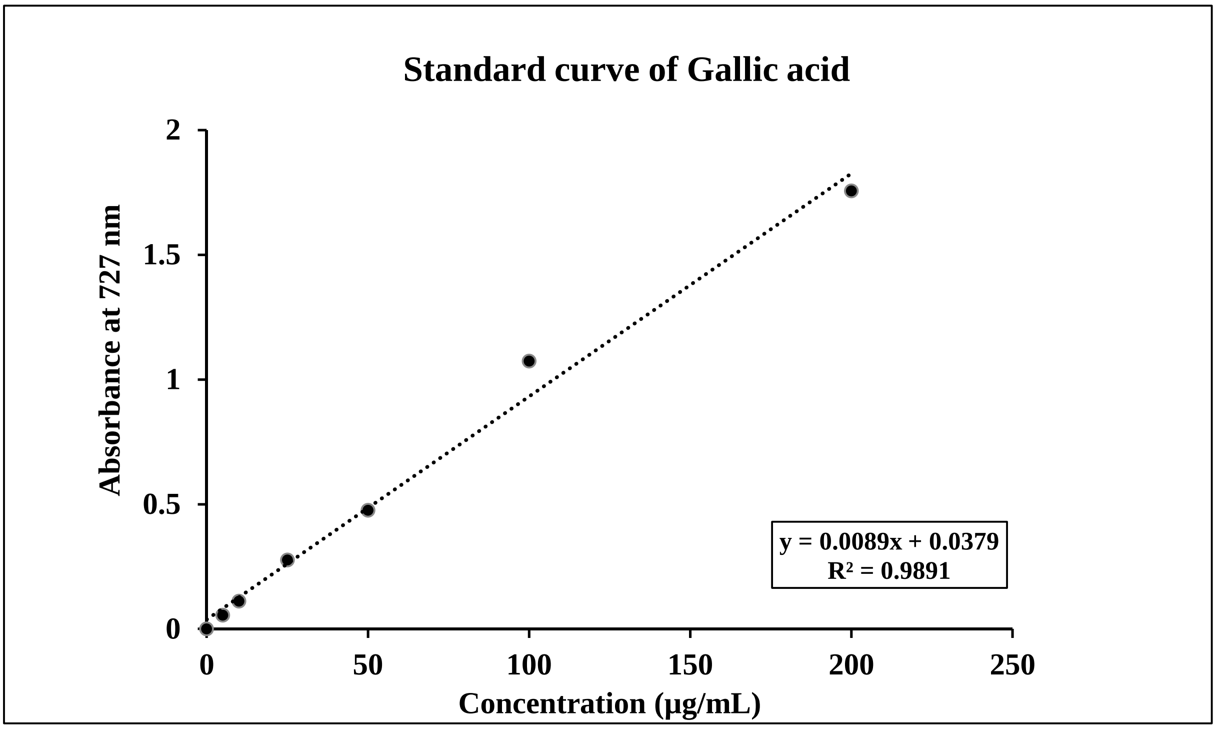

Figure S4. Calibration curve for Gallic acid (Standard).

References:

1. Okayo, R.O.; Andika, D.O.; Dida, M.M.; K'Otuto, G.O.; Gichimu, B.M. Morphological and Molecular Characterization of Toxigenic Aspergillus flavus from Groundnut Kernels in Kenya. *International Journal of Microbiology* **2020**, *2020*.
2. Nyongesa, B.W.; Okoth, S.; Ayugi, V. Identification key for Aspergillus species isolated from maize and soil of Nandi County, Kenya. *Advances in Microbiology* **2015**, *5*, 205.
3. Wickerham, L.J. Validation of the species Pichia guilliermondii. *Journal of bacteriology* **1966**, *92*, 1269.
4. Prameeladevi, T.; Prabhakaran, N.; Kamil, D.; Toppo, R.S.; Tyagi, A. Trichoderma pseudokoningii identified based on morphology was re-identified as T. longibrachiatum through molecular characterization. *Indian phytopathology* **2018**, *71*, 579-587.
5. Noonim, P.; Mahakarnchanakul, W.; Varga, J.; Frisvad, J.C.; Samson, R.A. Two novel species of Aspergillus section Nigri from Thai coffee beans. *International Journal of Systematic Evolutionary Microbiology* **2008**, *58*, 1727-1734.
6. Moubasher, A.; Soliman, Z. Aspergillus assiutensis, a new species from the air of grapevine plantation, Egypt. *Journal of Basic Applied Mycology, Egypt* **2011**, *2*, 83-90.
7. Zhao, R.-B.; Bao, H.-Y.; Liu, Y.-X. Isolation and characterization of Penicillium oxalicum ZHJ6 for biodegradation of methamidophos. *Agricultural Sciences in China* **2010**, *9*, 695-703.
8. Gomes, R.; Glienke, C.; Videira, S.; Lombard, L.; Groenewald, J.; Crous, P. Diaporthe: a genus of endophytic, saprobic and plant pathogenic fungi. *Persoonia: Molecular Phylogeny Evolution of Fungi* **2013**, *31*, 1.
